# Resting Energy Expenditure in Young Adults Born Preterm—The Helsinki Study of Very Low Birth Weight Adults

**DOI:** 10.1371/journal.pone.0017700

**Published:** 2011-03-25

**Authors:** Marika Sipola-Leppänen, Petteri Hovi, Sture Andersson, Karoliina Wehkalampi, Marja Vääräsmäki, Sonja Strang-Karlsson, Anna-Liisa Järvenpää, Outi Mäkitie, Johan G. Eriksson, Eero Kajantie

**Affiliations:** 1 Diabetes Prevention Unit, Department of Chronic Disease Prevention, National Institute for Health and Welfare, Oulu, Finland; 2 Department of Pediatrics and Adolescence, University of Oulu, Oulu, Finland; 3 Diabetes Prevention Unit, Department of Chronic Disease Prevention, National Institute for Health and Welfare, Helsinki, Finland; 4 Hospital for Children and Adolescents, Helsinki University Central Hospital and University of Helsinki, Helsinki, Finland; 5 Department of Obstetrics and Gynaecology, Oulu University Hospital, Oulu, Finland; 6 Department of Children, Young People and Families, National Institute for Health and Welfare, Oulu, Finland; 7 Department of General Practice and Primary Health Care, University of Helsinki, Helsinki Finland; 8 Vasa Central Hospital, Vasa, Finland; 9 Folkhälsan Research Institute, Helsinki, Finland; 10 Unit of General Practice, Helsinki University Central Hospital, University of Helsinki, Helsinki, Finland; University of Las Palmas de Gran Canaria, Spain

## Abstract

**Background:**

Adults born preterm with very low birth weight (VLBW; <1500g) have higher levels of cardiovascular and metabolic risk factors than their counterparts born at term. Resting energy expenditure (REE) could be one factor contributing to, or protecting from, these risks. We studied the effects of premature birth with VLBW on REE.

**Methodology/Principal Findings:**

We used indirect calorimetry to measure REE and dual x-ray absorptiometry (DXA) to measure lean body mass (LBM) in 116 VLBW and in 118 term-born control individuals (mean age: 22.5 years, SD 2.2) participating in a cohort study. Compared with controls VLBW adults had 6.3% lower REE (95% CI 3.2, 9.3) adjusted for age and sex, but 6.1% higher REE/LBM ratio (95% CI 3.4, 8.6). These differences remained similar when further adjusted for parental education, daily smoking, body fat percentage and self-reported leisure time exercise intensity, duration and frequency.

**Conclusions/Significance:**

Adults born prematurely with very low birth weight have higher resting energy expenditure per unit lean body mass than their peers born at term. This is not explained by differences in childhood socio-economic status, current fat percentage, smoking or leisure time physical activity. Presence of metabolically more active tissue could protect people with very low birth weight from obesity and subsequent risk of chronic disease.

## Introduction

Approximately 0.9 to 1.5% of all live-born infants in high-income countries are born preterm with very low birth weight (VLBW, <1500 g) [1], [2]. Advances in neonatal intensive care from the 1970s onwards have led to remarkable improvements in their survival [3], [4]; the first infants that have benefited from these advances are now young adults. It has recently become increasingly clear that many chronic adult diseases have their origins in intrauterine and early postnatal life [5], [6], [7], [8], [9]. VLBW infants experience conditions that are highly different from normal growth *in utero* and could therefore be at particularly increased risk. Accordingly, as adults they have substantially increased risk factors for chronic disease, such as impaired glucose regulation and up to 10 mmHg higher systolic blood pressure than their peers born at term [10], [11], [12], [13], [14].

Despite these cardiometabolic risk factors, VLBW adults are on average no more obese than those born at term: in previous studies adults born with VLBW rather tend to have a lower BMI [11], [15]. However, based on body composition measurements this difference is attributable to lower lean body mass (LBM) in VLBW adults, while there is little if any difference in fat mass [11]. As LBM is closely related to resting energy expenditure (REE) [16], adults born with VLBW would be expected to have lower REE. Further, as REE accounts for at least two-thirds of total energy expenditure, they would also be expected to have higher rates of obesity, which is not the case. One possibility is that VLBW adults have metabolically more active LBM, a phenomenon which has been shown in older adults who were born with a birth weight at the lower end of normal birth weight distribution [16], [17]. With this background, our primary aim was to study the effects of preterm birth with VLBW on REE in young adults. Our secondary aim was to assess whether these effects depend on perinatal conditions associated with preterm birth.

## Methods

### Participants

**Figure 1:**
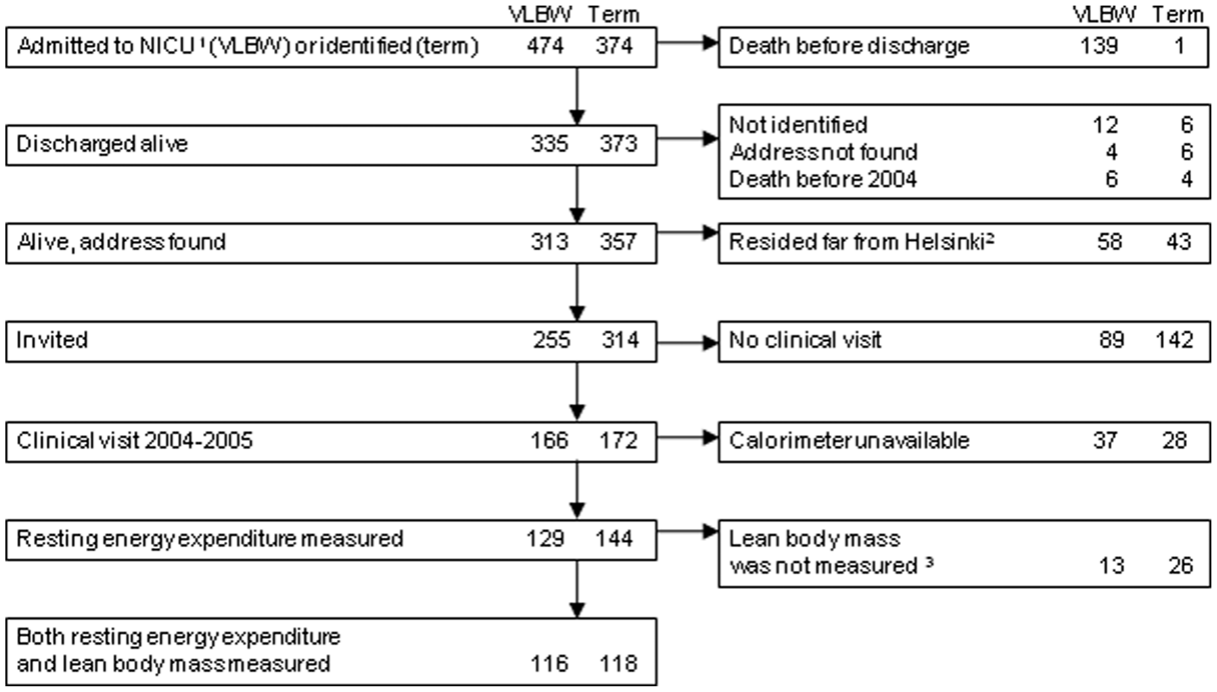
Flow chart showing participants selected for the present study. Participants who had both resting energy expenditure and lean body mass measured had similar characteristics compared to those invited but who did not undergo these measurements. ^1^NICU denotes neonatal intensive care unit. Term subjects were indentified from the birth-hospital records for each very low birth weight (VLBW) infant. ^2^Only those residing within distance of 110 km were invited. ^3^Lean body mass was not measured, if the subject was pregnant, had foreign object in the body, had severe cerebral palsy or was unwilling to undergo the examination.

This study is a part of the Helsinki Study of Very Low Birth Weight Adults, the details of which have been described [11], [14], [18]. Briefly, the original study cohort consisted of 335 infants who were born with VLBW between January 1978 and December 1985 and were discharged alive from the neonatal intensive care unit of Children's Hospital at Helsinki University Central Hospital, the only tertiary neonatal care center in the province of Uusimaa, Finland (Figure 1.). We selected a comparison group from the charts of all consecutive births at their birth hospitals. For each VLBW survivor, we selected the next available singleton term infant (gestational age >37 weeks), who was of the same sex and was not small for gestational age (birth weight more than - 2 SD).

We traced 95.1% of VLBW and 96.8% of controls through the National Population Register Center of Finland. We invited the 255 VLBW individuals and 314 control individuals born at term who were living in the greater Helsinki area to the first clinical visit to assess their adult health. 166 of the VLBW individuals (65.1%) and 172 of the control individuals (54.8%) agreed to participate. As previously reported in a detailed nonparticipation analysis, the perinatal and neonatal data (birth weight, length of gestation, maternal preeclampsia, days at discharge from the neonatal intensive care unit) for the clinical study participants and non-participants were similar, except for the lower rate of cerebral palsy among participants at 15 months of age [11]. REE could be measured for three of the four of five daily participants because there was only one calorimeter available. Those participants were selected randomly and no one refused. LBM required a separate visit and was not measured in participants who were pregnant or had a foreign object in the body, or had severe cerebral palsy or were unwilling to undergo the examination. As a result, 116 VLBW individuals and 118 controls born at term had data for both indirect calorimetry and body composition and were included in this study. These subjects did not differ from the remaining subjects who attended the clinical examination in any of the prenatal and birth characteristics (all p-values >0.30), except they were less likely to be born from a multiple pregnancy (p = 0.046). In addition, they were of similar age and had similar height, BMI, and parental education; they were also as likely to smoke and as likely to be male (all p-values >0.071).

### Perinatal and neonatal data

Perinatal and neonatal data were collected from hospital records. The infants born with VLBW had been weighed daily during their hospital stay and during clinical visits. If the weight at 40 weeks of gestational age was missing from the records, we included the interpolated value based on measurements available in within 10 days before and 20 days after this time point in our analysis. Weights were converted into standard deviation scores according to Finnish birth weight charts [19].

### Clinical data

At the mean age of 22.5 y (SD 2.2), range from 18.5 to 27.0, the participants attended a clinical examination which was performed in the clinic at the National Institute for Health and Welfare (formerly National Public Health Institute) after an overnight fast of at least 8 hours [11]. Height and weight were measured and body mass index (BMI) was calculated. Waist and hip circumferences were measured with a soft tape, waist circumference midway between the lowest rib and the iliac crest and hip circumference at the level of the great trochanters. In all of these measurements the participant was in underwear. The participants completed a detailed questionnaire on medical history, use of medication, current smoking, childhood socio-economic status (for which we used parents' education, categorized into four levels according to the parent with the higher education) and leisure-time physical activity [11](assessed with questions on exercise intensity (four categories), duration and frequency) [20].

### Resting Energy Expenditure and Lean Body Mass

The main outcome variable was REE, which was measured with indirect calorimetry (Deltatrac II, Datex, Helsinki, Finland) at rest. Indirect calorimetry is the most commonly used method to measure REE, and Deltatrac has been well-established as a valid and reliable device [21], [22], [23]. The device uses a computerized, open-circuit system to measure gas exchange through a transparent plastic canopy, which covers the head of the participant. Flow was measured by the air-dilution method. The measurement was performed by one of two trained nurses with the subject wearing light indoor clothing. The measurement was performed after the subject had come to the clinic after an overnight fast, had completed the consent forms and had undergone the anthropometric and blood pressure measurements, usually approximately 30 minutes after coming to the clinic. The subject was first connected to the device and rested about 10 minutes before the measurement started. During the measurement the subject, still fasting, was lying in a bed in a comfortable semi-recumbent position. REE was expressed as as the amount of energy used in 24 h [16], [24], [25], [26], [27], [28].

LBM was measured with whole body dual energy x-ray absorptiometry (DXA, Hologic® Discovery A, software version 12.3∶3, Bedford, MA, USA) as described [11], [18]. DXA is found to be reliable and reproducible in the estimation of LBM, although it does not distinguish between visceral and subcutaneous fat tissue [29], [30], [31]. Subjects were wearing underwear and were asked to remove all jewelry and other personal effects that could interfere with the DXA measurement. Measurements that contained artifacts which could affect the accuracy of the DXA results were set to missing in the dataset. The manufacturer's software was used to separate lean body mass (LBM) and fat mass (FM) We calculated the ratio of REE and LBM (REE/LBM) [16].

### Ethics

The study was performed according to the Declaration of Helsinki, and its protocol was approved by the Ethics Committee for Children's and Adolescents' Diseases and Psychiatry of the Helsinki and Uusimaa Hospital District. Each participant gave a written informed consent.

### Statistical methods

All statistical analyses were performed with SPSS for Windows, Version 16.0. Outcome variables (REE, LBM, REE/LBM ratio) with skewed distributions were normalized using logarithmic transformation. Crude group differences were assessed by use of the t-test or χ^2^-test. We used linear regression to adjust for covariates (age, sex, parental education, daily smoking, body fat percentage and the self-reported intensity, frequency and duration of leisure time conditioning physical activity).

## Results

Characteristics of the study population are shown in Table 1 and Table 2. As compared with controls, VLBW adults were shorter, had lower BMI and lower lean body mass, but similar body fat percentage [11].

**Table 1:** Prenatal and birth characteristics of very low birth weight (VLBW, <1500g) infants and term born controls; numbers represent mean (SD, standard deviation) or n (%).

	VLBWn = 116	Controls n = 118	p-value (t test)
**Birth weight (g)**	1125 (223)	3606 (469)	<0.001
**Gestational age (weeks)**	29.2 (2.3)	40.1 (1.1)	<0.001
**Small for gestational age, n (%)**	25 (21.6)	0 (0)	N/A
**Mother**'**s preeclampsia, n (%)**	24 (20.7)	10 (8.5)	0.008
**Multiple pregnancies, n (%)**	21 (18.1)	0 (0)	N/A

*The characteristics were compared using the t-test.*

N/A = *not applicable.*

**Table 2:** Clinical characteristics of the young adults born with very low birth weight (VLBW; <1500 g) and term born controls; the numbers represent mean (SD, standard deviation) or n (%).

Characteristics	Sex	VLBW	Term	P-value
**Participants, n**	**-**	116	118	
**Males, n (%)**	**-**	44 (37.9)	45 (38.1)	0.97*
**Age at clinical examination (years)**	**-**	22.3 (2.2)	22.6 (2.2)	0.42
**Current weight (kg)**	F	58.2 (12.3)	64.9 (11.3)	<0.001
	M	69.0 (14.5)	78.8 (11.8)	<0.001
**Current height (cm)**	F	162.4 (8.0)	167.6 (6.3)	<0.001
	M	175.9 (8.5)	180.7 (6.1)	0.003
**Body mass index (kg/m2)**	F	21.8 (3.7)	23.0 (3.9)	0.064
	M	22.1 (4.0)	23.9 (3.2)	0.020
**Body fat percentage**	F	31.9 (6.2)	32.0 (5.6)	0.92
	M	19.9 (6.4)	19.9 (5.4)	0.97
**Lean body mass, LBM (kg)**	F	38.9 (5.9)	43.5 (5.5)	<0.001
	M	54.4 (8.9)	62.2 (8.4)	<0.001
**Resting energy expenditure, REE (kcal/24 h)**	F	1442 (207)	1520 (172)	0.014
	M	1834 (253)	1977 (237)	0.008
**REE/LBM ratio (kcal/24 h/kg)**	F	37.4 (4.4)	35.1 (3.0)	<0.001
	M	33.9 (2.6)	31.9 (2.3)	<0.001
**Daily smoking (yes/no), n (%)**				0.27*
No	**-**	90 (78.3)	85 (72.0)	
Yes	**-**	25 (21.7)	33 (28.0)	
**Educational level of the more educated parent, n(%)**				0.029†
Elementary	**-**	11 (9.6)	8 (6.8)	
High school	**-**	30 (26.1)	24 (20.3)	
Intermediate	**-**	41 (35.6)	33 (28.0)	
University	**-**	33 (28.7)	53 (44.9)	
**Self-reported intensity of leisure time conditioning physical activity, n (%)**				<0.001†
Walking	**-**	33 (28.7)	14 (11.9)	
Intermittent walking and light running	**-**	33 (28.7)	31 (26.3)	
Light running (jogging)	**-**	32 (27.8)	30 (25.4)	
Brisk running	**-**	17 (14.8)	43 (36.4)	
**Self-reported frequency of leisure time conditioning physical activity**				0.43†
Not at all	**-**	4 (3.5)	3 (2.5)	
Less than once a month	**-**	14 (12.2)	12 (10.2)	
1-2 times a month	**-**	13 (11.3)	7 (5.9)	
Approximately once a week	**-**	26 (22.6)	29 (24.6)	
2 to 3 times a week	**-**	30 (26.1)	38 (32.2)	
4 to 5 times a week	**-**	14 (12.2)	18 (15.3)	
About daily	**-**	14 (12.2)	11 (9.3)	
**Self-reported average duration of leisure time physical activity session**				<0.001†
< 30 minutes or no exercise	**-**	16 (13.9)	6 (5.1)	
30 minutes to <1 hour	**-**	46 (40.0)	26 (22.0)	
1 hour to <2 hours	**-**	48 (41.7)	74 (62.7)	
≥2 hours	**-**	5 (4.3)	12 (10.2)	

*The characteristics were compared using the t-test, unless otherwise indicated;*

*chi-square-test,

† *p for linear trend. F = female, M = male.* N/A* = not applicable.*

### Resting Energy Expenditure and Lean Body Mass

**Figure 2:**
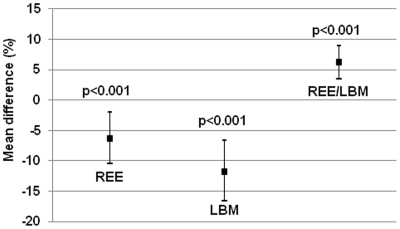
Mean difference between VLBW and term born young adults: linear regression models. Mean difference in percent in resting energy expenditure (REE), lean body mass (LBM) and the proportion of REE to LBM (REE/LBM) between the very low birth weight (VLBW) (error bars showing 95% confidence intervals) and control groups (zero line) in linear regression model adjusted for age and sex.

Table 3 and Figure 2 show that both women and men with VLBW had lower REE than their counterparts born at term. However their REE/LBM ratio was higher. There was no interaction between the effects of VLBW birth and sex on these outcomes (all p values >0.27), meaning that the relationship between prematurity and REE was similar in men and women, and therefore we present the results pooled for both sexes. The differences in REE and REE/LBM ratio were little affected by adjustment for age, sex, parental education, daily smoking, body fat percentage and the intensity, frequency and duration of self-reported leisure time physical activity (Table 3).

**Table 3:** Linear regression models showing differences in resting energy expenditure (REE) and the proportion of REE to lean body mass (REE/LBM ratio) (95% confidence intervals) between VLBW and term born young adults, unadjusted and adjusted for covariates in different models.

Model	N	REE	REE/LBM ratio
Unadjusted	234	−6.3% (−10.4 to −2.0)	6.2% (3.5 to 9.0)
1	234	−6.3% (−9.3 to −3.2)	6.1% (3.4 to 8.6)
2	233	−6.3% (−9.4 to −3.2)	6.0% (3.5 to 8.5)
3	233	−4.7% (−7.7 to −1.6)	5.7% (3.2 to 8.4)

*Model 1: Adjusted for age and sex,*

*Model 2: Adjusted for 1+ parental education (4 levels) and daily smoking,*

*Model 3: Adjusted for 2+ body fat percentage and the self-reported intensity, frequency and duration of leisure time physical activity.*

We reanalyzed the data after exclusion of subjects born from multiple pregnancies (21 of VLBW, 0 controls), subjects with cerebral palsy, developmental delay, severe sensorineural deficit (a total 12 of VLBW individuals and 1 control) or with regular usage of beta-sympathomimetic drugs (3 VLBW individuals, 3 controls). The results were similar.

### Effects of perinatal factors and other clinical characteristics associated with preterm birth

The 25 VLBW adults born small for gestational age (SGA) had 6.2% (95% CI −11.5% to 0.15%, p = 0.056) lower REE than the 91 VLBW adults born appropriate for gestational age (AGA) when adjusted for age and sex. The difference attenuated after further adjustment for other covariates. There was no difference in REE/LBM ratio between these groups; their mean difference was 0.6% (95% CI −3.9% to 5.2%, p = 0.81). Within the VLBW group, individuals whose mothers had preeclampsia during pregnancy, as compared to those whose did not, had similar REE (mean difference 3.8%; 95% CI −2.6% to 10.5%, p = 0.24) and similar REE/LBM ratio (mean difference −0.7%; 95% CI −5.1% to 3.8%, p = 0.74).

In the VLBW group, body weight at what would have been term (40 weeks of postmenstrual age) could be determined for 81 of 116 subjects. The mean (SD) score for weight at term was −2.6 (1.2) and the mean change from birth to term was −1.4 (1.3). A 1 standard-deviation unit higher score corresponded to 3.3% increase in REE (95% CI 0.6% to 6.1%, p = 0.019) and was not related to REE/LBM ratio (p = 0.13) when adjusted for age and sex. The chance in standard deviation score from birth to term was not related to REE (p = 0.87) or REE/LBM ratio (p = 0.25).

## Discussion

We found that, although adults born preterm with VLBW had lower REE than their peers born at term, they had higher REE per unit LBM. The difference was not explained by differences in body fat percentage, smoking, childhood socio-economic status or self-reported leisure time physical activity. The higher REE/LBM ratio was seen in both sexes, and it seemed to be associated with VLBW birth *per se* rather than any related perinatal conditions. This finding is in accordance with previous studies in older people born with a low-normal birth weight [16], [17] and extends them by showing that in people born with VLBW this phenomenon is observed in young adulthood.

LBM explains about 60% of the variation of REE between individuals [32]. In accordance with previous studies [16], [17], [32], we found that the lower REE in VLBW adults is largely attributable to their lower LBM. However, the higher REE/LBM ratio in VLBW adults implies that they may have more metabolically active tissue than the controls. This may be counterintuitive, because studies of organ size have suggested that the metabolically most active organs are particularly small in VLBW adults. For example, as compared with skeletal muscle, kidney has a 30-fold and brain a 20-fold higher resting metabolic rate per gram tissue [33]. In a Dutch study, VLBW adults had smaller kidneys in relation to their smaller body size than controls born at term, although selection bias and confounding remain possible in that study that used controls recruited as adults by advertisements. The smaller size of the brain is a consistent finding in VLBW adolescents and adults [34], [35] (which account for about 60% of the REE in adults in general [26]. DXA and calorimetry are unable to distinguish whether the differences in REE/LBM ratio are due to higher metabolic rate of some specific organ or an overall increase in metabolic rate in adults born with VLBW. Such an increase could be contributed to by increased sympathetic nervous system activity, although existing data are not consistent. Adults born with VLBW have a higher heart rate suggesting an elevated cardiac sympathetic drive [11], whereas other studies suggest a lower muscle sympathetic nerve activity [36].

Studies have shown that fat mass or adipose tissue explains a small part of individual variation on REE [32], [37], [38]. In the present study cohort, however, there was no difference in body fat percentage or fat distribution between VLBW and control groups [11]. Moreover, it has recently been shown that brown adipose tissue, which is involved in non-shivering thermogenesis (heat production in response to environmental temperature or diet) and is thus metabolically active, can be present in adulthood [39]. The presence and amount of brown adipose tissue could in theory underlie the higher REE/LBM in VLBW compared to control subjects, but its detection would have required advanced Positron emission tomography (PET) technology, which was not available in our study.

REE accounts for about two thirds of daily total energy expenditure (TEE). The remainder of TEE depends on individual's thermogenesis and physical activity, the amount of which varies substantially between individuals [40]. Although REE is a remarkably stable measurement, with intraindividual variability ranging from 3% to 7.5% [23], there are individual differences in resting metabolic rate. Follow-up studies [41] and studies of formerly obese individuals [42] suggest that these differences could be important contributors to the development of obesity. A consistent finding in previous studies is lower rates of leisure-time physical activity in VLBW compared to term-born control young adults [11], [18], [20], [43], [44], [45], [46]. This underlies the significance of REE in TEE as a factor protecting from obesity in people born with VLBW. Previous studies have concluded that promotion of physical activity should be incorporated in the follow-up of children born preterm. In addition to more direct benefits, physical activity is also expected to increase LBM and thereby REE.

We observed little difference in REE or REE/LBM ratio between VLBW young adults born SGA compared with those born AGA. Consistent with this, we have previously shown that SGA and AGA VLBW subjects have similar levels of cardiovascular risk factors including impaired glucose regulation [11] and high blood pressure [11], [14], [47], both increased as compared with those born at term. Although, by definition, the VLBW-SGA and VLBW-AGA groups differ in the conditions experienced before birth, they have a similar experience after preterm birth. It is therefore possible that the long-term effects of adverse conditions during the period after preterm birth override those related to the conditions leading to preterm birth such as intrauterine growth restriction. However, this should be interpreted with caution since our study has limited power for subgroup analyses such as those for SGA and AGA. Nevertheless, that early growth pattern is important is also supported by our finding that the change in weight standard deviation score from birth to term was not related to REE or REE/LBM ratio, although a higher weight attained at 40 postmenstrual weeks (term) was related to higher REE through its association with LBM.

### Study Limitations

We have previously discussed the limitations of the Helsinki Study of Very Low Birth Weight Adults [11], [48]. Although our participation rates were similar to most other VLBW follow-up studies [12], [13], [46], [49], not all participants underwent calorimetry and DXA. Although there was little difference in background characteristics between these participants and those who did not undergo these studies, participation bias cannot be excluded. This, however, would be expected to affect the results only if the association between VLBW birth and resting energy expenditure was different in participants and non-participants. This is unlikely but cannot be excluded. Moreover, we had no direct measurement of visceral fat or brown adipose tissue which may be important contributors to energy metabolism [39].

### Conclusion

In conclusion, young adults born with VLBW have a higher ratio of REE to LBM than their peers born at term. This suggests that they have metabolically more active lean tissue. While our results add to previous findings of early programming of metabolic characteristics, they also suggest that some of these characteristics may be protective for example in terms of preventing obesity.
